# Ocular Blood Flow in Eyes with Myopia and Glaucoma: From Pathophysiology to Clinical Implications

**DOI:** 10.3390/jcm15166291

**Published:** 2026-08-14

**Authors:** Naoki Takahashi, Naoki Kiyota, Akiko Hanyuda, Satoru Tsuda, Toru Nakazawa

**Affiliations:** 1 Department of Ophthalmology, Tohoku University Graduate School of Medicine, Sendai 980-8574, Miyagi, Japan; naoki.takahashi.a4@tohoku.ac.jp (N.T.);; 2 Feinberg Cardiovascular and Renal Research Institute and Division of Nephrology and Hypertension, Northwestern University Feinberg School of Medicine, Chicago, IL 60611, USA; 3 Department of Ophthalmology, Keio University School of Medicine, Shinjuku 160-8582, Tokyo, Japan; 4 Division of Ophthalmic Precision Medicine Development, United Centers for Advanced Research and Translational Medicine (ART), Tohoku University Graduate School of Medicine, Sendai 980-8575, Miyagi, Japan; 5 Department of Retinal Disease Control, Tohoku University Graduate School of Medicine, Sendai 980-8574, Miyagi, Japan; 6 Department of Advanced Ophthalmic Medicine, Tohoku University Graduate School of Medicine, Sendai 980-8574, Miyagi, Japan

**Keywords:** myopic glaucoma, myopic optic neuropathy, glaucomatous optic neuropathy, ocular blood flow, normal-tension glaucoma, OCT angiography, laser speckle flowgraphy, choroidal microvasculature dropout, lamina cribrosa, central visual field damage

## Abstract

The global prevalence of myopia is increasing, and the combination of myopia and glaucoma is widely expected to become an important clinical and public health issue. Myopic eyes undergo axial elongation and posterior pole remodeling, leading to optic disc tilt, peripapillary deformation, choroidal thinning, and altered spatial relationships between the optic nerve head and its vascular supply. Glaucomatous optic neuropathy is associated with retinal ganglion cell damage, lamina cribrosa deformation, and ocular blood flow impairment. Recent advances in optical coherence tomography angiography and laser speckle flowgraphy have enabled noninvasive assessment of retinal, peripapillary, choroidal, and optic nerve head circulation. These modalities have revealed reduced radial peripapillary capillary density, choroidal microvasculature dropout, and reduced optic nerve head tissue blood-flow signal in glaucomatous eyes. In eyes with myopia and glaucoma, myopia-related structural deformation and glaucoma-associated perfusion abnormalities may coexist and show sectoral correspondence with neural damage, particularly in regions related to the central visual field. This review summarizes the anatomical background of ocular blood flow, myopia-induced structural and vascular changes, glaucoma-associated blood flow impairment, and their interaction in eyes with myopia and glaucoma. Potential therapeutic approaches targeting ocular blood flow are also discussed, although evidence beyond intraocular pressure reduction remains limited.

## 1. Introduction

Myopia is becoming more prevalent worldwide, increasing the associated public health challenges, particularly in East Asia. Holden et al. estimated that by 2050, approximately half of the world’s population may be affected by myopia and nearly 10% by high myopia [1]. Myopia is not merely a refractive error; myopic axial elongation causes remodeling of posterior pole tissues, choroidal thinning, and deformation of peripapillary structures, exerting multifaceted effects on the retina, choroid, and optic nerve head [2]. These changes may also contribute to the development and progression of glaucomatous optic neuropathy (GON).

Myopia is an important risk factor for open-angle glaucoma. A meta-analysis of observational studies showed that myopic eyes have a higher risk of developing open-angle glaucoma than non-myopic eyes, and that the risk increases as myopia becomes more severe [3]. Recent longitudinal studies have also reported high myopia as a major risk factor for the development of open-angle glaucoma [4]. In myopic eyes, optic disc tilt, peripapillary atrophy (PPA), displacement and enlargement of the Bruch’s membrane opening (BMO), and thinning or deformation of the lamina cribrosa may occur. These changes increase mechanical stress on the optic nerve head and collectively make the diagnosis of glaucoma more challenging [2].

Traditionally, the pathogenesis of glaucoma has been understood mainly in terms of intraocular pressure (IOP)-related deformation of the lamina cribrosa and subsequent injury to retinal ganglion cell axons [5]. Indeed, IOP reduction remains the most established therapeutic intervention for slowing glaucoma progression, and its efficacy was demonstrated in the Early Manifest Glaucoma Trial [6]. However, in Asia, including Japan, normal-tension glaucoma (NTG) accounts for a large proportion of glaucoma cases [7]. In fact, the Tajimi Study reported that the majority of eyes with open-angle glaucoma had an IOP of 21 mmHg or lower [8]. This suggests that factors other than IOP, particularly optic nerve head perfusion, vascular dysregulation, low ocular perfusion pressure, and systemic vascular factors, may be involved in the pathophysiology of glaucoma [9,10,11,12,13,14].

**Figure 1:**
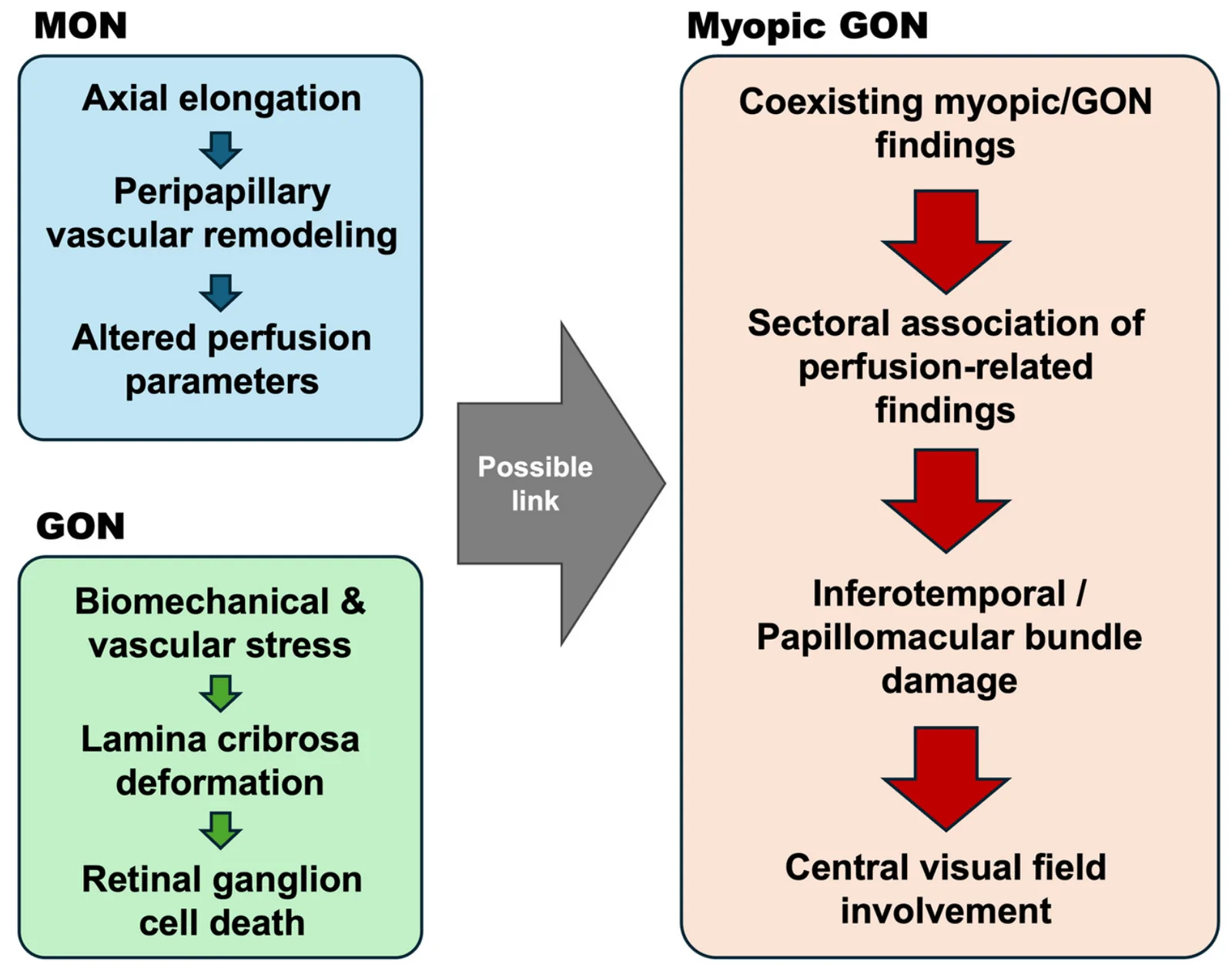
Proposed framework for structural and perfusion-related findings in eyes with coexisting myopia and glaucoma. In this framework, co-occurring myopia and glaucoma are characterized by an overlap between myopic optic neuropathy (MON) and glaucomatous optic neuropathy (GON). Myopia-induced structural changes, such as axial elongation, optic disc tilt, peripapillary deformation, and peripapillary vascular displacement, coexist with glaucoma-associated perfusion-related abnormalities and may show sectoral correspondence with neural damage, including in regions related to central visual function.

Thus, to understand the pathogenesis of glaucoma in eyes with myopia, it is necessary to integrate an understanding of myopia-related structural changes in the optic nerve head and peripapillary tissues and view the problem from the perspective of ocular circulation and microvascular impairment. However, comprehensive reviews that systematically address myopia, ocular blood flow, and GON remain limited. Thus, in this review, we summarize reports on myopia-related structural changes in the posterior pole, the anatomical background of the peripapillary circulation, optical coherence tomography angiography (OCTA)-derived perfusion-related parameters, laser speckle flowgraphy (LSFG)-derived hemodynamic indices, and the clinical significance of ocular circulatory abnormalities in eyes with myopia and glaucoma (Figure 1). We further discuss the role of vascular factors in understanding the pathophysiology of the combination of myopia and glaucoma.

## 2. Methods

This narrative review was informed by searches of PubMed/MEDLINE from database inception through June 2026. Broad combinations of the following terms were used: “myopia,” “high myopia,” “myopic optic neuropathy,” “glaucoma,” “normal-tension glaucoma,” “ocular blood flow,” “ocular perfusion,” “optic nerve head blood flow,” “optical coherence tomography angiography,” “OCTA,” “vessel density,” “microvasculature dropout,” “laser speckle flowgraphy,” and “LSFG.” Targeted combinations with “peripapillary,” “choroid,” “lamina cribrosa,” “central visual field,” “artifact,” and “treatment” were added when relevant. Reference lists of pertinent articles were also screened. Because this was a narrative review, study selection was guided by relevance to myopia-associated structural remodeling, glaucomatous optic neuropathy, ocular perfusion, imaging methodology, and clinical interpretation rather than by a formal systematic-review protocol. ChatGPT (version 5.5) was used solely to assist with literature searching and identification of potentially relevant references; all cited references were independently verified by the authors.

## 3. Anatomical Background of Ocular Blood Flow

A clear understanding of the vascular anatomy of the optic nerve head and peripapillary tissues is essential for interpreting alterations in ocular blood flow in myopia and glaucoma. Ocular blood flow is supplied primarily by the ophthalmic artery, a branch of the internal carotid artery, through several major vascular systems, including the retinal, choroidal, and optic nerve head circulations. Among the branches in this system, the central retinal artery (CRA) and posterior ciliary arteries (PCAs) play key roles in supplying the retina, choroid, and optic nerve head [15,16]. The CRA primarily supplies the inner retina, including the retinal nerve fiber layer (RNFL) and ganglion cell layer. The PCA system is the primary source of supply to the choroid and the deeper structures of the optic nerve head. Within the optic nerve head, the superficial nerve fiber layer is supplied mainly by the retinal circulation, whereas the lamina cribrosa is supplied predominantly by branches of the short PCAs (SPCAs), the peripapillary choroid, and the arterial circle of Zinn–Haller [17,18].

The PCAs are broadly divided into the long and short PCAs. The course of the long PCAs is anterior, and they are involved primarily in the anterior uveal circulation, supplying structures such as the iris and ciliary body. The SPCAs penetrate the sclera at the posterior pole and supply the choroid and peripapillary region [16]. There are multiple SPCAs, and their branches form segmental perfusion territories within the choroid. Accordingly, the choroidal and optic nerve head circulations do not constitute a completely homogeneous vascular bed; rather, watershed zones may form between SPCAs or between choroidal arteries [19]. This segmental organization of the PCA system provides an important anatomical basis for understanding why ischemic lesions of the optic nerve head and choroid often appear in a focal or sectoral pattern [19]. Hayreh et al. emphasized that the blood supply of the optic nerve head shows marked interindividual variability. This variability may reflect several anatomical and hemodynamic factors, including the number of PCAs, the size of the region supplied by each PCA, the location of watershed zones, and pressure differences within the PCAs and SPCAs [20]. Therefore, even under similar IOP and systemic blood pressure conditions, the susceptibility of the optic nerve head to ischemia may differ between individuals and may also vary across regions within the optic nerve head.

One important structure in the deep circulation of the optic nerve head is the circle of Zinn–Haller. The circle of Zinn–Haller is an arterial anastomosis located within the peripapillary sclera, derived mainly from branches of the SPCAs, and is thought to contribute to the blood supply of the deep optic nerve head, particularly the lamina cribrosa [18]. However, the morphology and continuity of this arterial circle vary between individuals, and it does not necessarily form a complete circular structure [21]. An indocyanine green angiography (ICGA) study of highly myopic eyes showed that the circle of Zinn–Haller is often only partially visible and may not function as a complete collateral pathway between the medial and lateral PCAs [22].

From a hemodynamic perspective, optic nerve head blood flow is influenced not only by the anatomical distribution of its vascular supply but also by IOP, systemic blood pressure, intravascular capillary pressure, and peripheral vascular resistance. Hayreh et al. identified IOP, mean capillary blood pressure, and peripheral vascular resistance, in addition to anatomical patterns of vascular supply, as factors contributing to interindividual variability in optic nerve head blood flow [20]. Therefore, when ocular perfusion pressure is reduced due to elevated IOP or low systemic blood pressure, regional characteristics of the PCA system, the location of watershed zones, and a circle of Zinn–Haller that is not completely continuous may together render specific regions of the optic nerve head relatively more vulnerable to ischemia [23].

Thus, the optic nerve head circulation represents a complex vascular system composed of the superficial retinal circulation, supplied by the CRA, and the deeper circulation, supplied by the SPCAs, peripapillary choroid, pial vessels, and the circle of Zinn–Haller. In particular, the lamina cribrosa and its surrounding tissues are major sites of glaucomatous optic nerve damage. The substantial dependence of this region on the PCA-derived circulation provides an important anatomical basis linking IOP-dependent mechanical stress with IOP-independent vascular factors.

## 4. Imaging Assessment Using LSFG and OCTA

Traditionally, fundus blood flow has been evaluated using fluorescein angiography and ICGA. These techniques are useful for assessing perfusion abnormalities in the retinal and choroidal circulations; however, because they require intravenous dye injection, they are limited by their invasiveness and reduced suitability for repeated measurements. In recent years, noninvasive imaging modalities such as LSFG and OCTA have been introduced into clinical practice, enabling more convenient and repeatable assessment of ocular fundus circulation.

### 4.1. OCTA

OCTA is an imaging technique that visualizes vascular structures by analyzing signal changes between repeated optical coherence tomography (OCT) B-scans acquired at the same location, thereby deriving motion contrast from the movement of red blood cells. A major advantage of OCTA is that it enables three-dimensional and layer-by-layer assessment of the retinal and peripapillary microvasculature without the need for intravenous dye injection [24]. In the field of glaucoma, the parameters most often reported are vessel density in the radial peripapillary capillary (RPC) slab and focal flow-signal deficits in deeper peripapillary slabs [25]. Slab labels are device-specific, and the same label does not always represent the same anatomy: parapapillary “deep-layer” microvasculature dropout, for example, was originally assessed on the choroidal-layer vessel density map rather than within a retinal plexus [26]. In this review, “deep peripapillary” therefore denotes the device-defined slab reported in the cited study, and anatomical structures such as the peripapillary choroid or the deep optic nerve head tissue are named explicitly when a histological concept is intended.

OCTA also does not directly quantify volumetric blood flow; it detects motion contrast from particles moving above a device-dependent velocity threshold. Slow flow or weak signal may therefore leave anatomically present vessels undetected, and motion, projection, and segmentation artifacts, optic disc tilt, and image magnification require particular attention in myopic eyes [25,27,28,29,30].

Beyond static vessel-density maps, OCTA can capture cardiac-cycle-dependent variation in flow signal. Episodic flow-related signals and layer-specific reperfusion patterns have been demonstrated following acute IOP elevation in a nonhuman primate model [31]. Eyes with markedly elevated IOP can show alternating hyporeflective and hyperreflective bands synchronized with arterial pulsation on OCTA scans, with recovery of measured vessel density after IOP lowering [32]. These observations indicate that the OCTA flow signal is time-dependent and threshold-limited.

### 4.2. LSFG

LSFG is a noninvasive imaging technique that evaluates ocular fundus blood flow by analyzing changes in the speckle pattern generated when laser light projected onto the fundus is scattered by moving red blood cells. The mean blur rate (MBR), a representative LSFG parameter, is considered to reflect the relative blood flow velocity within the measurement area. Although LSFG cannot depict capillary structures in detail as OCTA does, it has the advantage of enabling rapid and repeatable measurements of blood flow in the optic nerve head [33,34].

In addition, LSFG enables the calculation of waveform parameters, such as blowout score and blowout time, from pulse-synchronized blood flow waveforms, thereby allowing assessment not only of blood flow velocity but also of pulsatility and blood flow stability [35]. Reduced optic nerve head MBR has been reported in glaucomatous eyes, and associations with visual field damage and disease progression have also been suggested [36,37,38]. However, it should be noted that MBR may be influenced by factors such as IOP, systemic blood pressure, heart rate, refractive error, axial length, optic disc morphology, and imaging conditions [39].

### 4.3. Complementary Roles of LSFG and OCTA

OCTA and LSFG both enable noninvasive assessment of ocular fundus circulation, but their outputs are distinct and complementary. OCTA provides layer-specific perfusion-related motion-contrast information, including vessel density and flow-signal deficits, but does not directly quantify volumetric blood flow. In contrast, LSFG provides relative hemodynamic indices, including MBR and pulse-waveform parameters. Although OCTA- and LSFG-derived parameters may correlate, they do not represent identical aspects of ocular circulation. In eyes with myopia and glaucoma, their complementary use may support integrated assessment of microvascular architecture and relative hemodynamic change [40,41].

## 5. Myopia-Induced Structural and Vascular Changes

In myopia, remodeling of the posterior pole caused by axial elongation alters the morphology of the optic nerve head, peripapillary sclera, choroid, and retinal vessels. These changes are not merely consequences of refractive error; they influence the vascular course and perfusion environment around the optic nerve head. In particular, optic disc tilt, misalignment between the BMO and the clinically visible disc margin, PPA, and choroidal thinning are characteristic structural changes in myopic eyes [42]. These alterations may affect the retinal circulation, the perfusion of the deep optic nerve head tissue, and the choroidal circulation; however, many of them should be understood as changes due to myopic ocular deformation, independent of glaucomatous damage [43]. Therefore, this section focuses on structural changes and alterations in blood flow or vascular parameters induced by myopia itself, distinguishing them from blood flow impairment associated with GON.

### 5.1. Myopia-Induced Optic Disc Deformation and Peripapillary Vascular Displacement

In myopic eyes, axial elongation is accompanied by expansion of the posterior pole, resulting in characteristic peripapillary structural changes, such as optic disc tilt, torsion, and enlargement of the PPA area [44,45]. Recent OCT studies have refined the interpretation of PPA by distinguishing the β-zone, which contains Bruch’s membrane, from the γ-zone, which lacks Bruch’s membrane [46]. Whereas the β-zone is relatively more strongly associated with glaucomatous changes, the γ-zone is generally understood as a structural manifestation of myopic ocular expansion and misalignment between the BMO and the clinically visible disc margin.

These structural changes indicate that myopic optic disc deformation is not simply an enlargement or atrophy of peripapillary tissues, but a three-dimensional rearrangement of the optic nerve head and surrounding structures. In myopic eyes, the spatial relationships are altered among the BMO, scleral canal, peripapillary sclera, and choroid [47]. Accordingly, the peripapillary vascular architecture may not simply undergo atrophic change, but may also be displaced or redistributed in association with ocular expansion. Chan et al. interpreted the myopic tilted disc as a three-dimensional deformation of the optic nerve head with reference to the BMO, and they emphasized that evaluation of peripapillary structures in myopic eyes should incorporate investigation with OCT of the BMO and PPA in addition to fundus photography observation of the disc margin [42].

Furthermore, even in otherwise healthy myopic eyes, focal microvascular signal defects may be observed in OCTA images in the distal PPA region. Kim et al. reported that non-juxtapapillary microvasculature dropout (MvD) was observed in approximately 20% of otherwise healthy myopic eyes without glaucoma, and that most of these lesions were associated with misalignment of the Bruch’s membrane–retinal pigment epithelium (BM–RPE) complex or enlargement of the γ-zone [48]. This finding should be distinguished from peripapillary choroidal MvD (CMvD) within the β-zone, which is typically observed in glaucomatous eyes, and may reflect microvascular signal defects associated with myopic stretching of peripapillary tissues [48]. Therefore, when vascular signal defects are observed in OCTA images in myopic eyes, they should not immediately be interpreted as MvD derived from GON. Instead, their relationship with myopia-related structural changes, such as the γ-zone, BMO misalignment, and optic disc tilt, should be carefully assessed.

### 5.2. Retinal Circulation and Central Retinal Artery-Related Changes

Narrowing of the retinal vessels and reduced retinal blood flow have been reported in myopic eyes. Studies in children and young adults have also suggested that retinal vessel caliber and vascular geometric parameters may change with increasing axial length and greater myopic refractive error [49,50]. However, because axial elongation alters the magnification of fundus images, apparent changes in vessel caliber or vessel density should be interpreted with caution. Yii et al. reported that after magnification correction for axial length, retinal vessel narrowing associated with myopia could be explained largely by optical magnification effects [51].

Studies using OCTA have yielded inconsistent results regarding changes in peripapillary capillary density in myopic eyes. Suwan et al. evaluated peripapillary perfused capillary density in eyes with and without myopia or glaucoma, and reported that the reduction in vessel density was relatively minor in eyes with myopia alone, whereas the presence of glaucoma had a greater effect [52]. In contrast, Mo et al. reported reduced macular and peripapillary vascular flow density in eyes with pathologic myopia, suggesting that such changes may occur in these eyes or in those with advanced posterior pole deformation [53]. Taken together, these findings suggest that observations of retinal circulatory changes in myopia may be strongly influenced by optical magnification and analytic methods in mild to moderate myopia, whereas true reductions in vessel density or perfusion may occur in eyes with pathologic myopia or marked axial elongation. Therefore, magnification correction and structural assessment are essential when interpreting OCTA findings in myopic eyes [27].

### 5.3. Deep Optic Nerve Head Circulation

In contrast to the superficial retinal circulation, the deep circulation of the optic nerve head is supplied mainly by the PCA-derived vascular system, including the SPCAs, peripapillary choroid, and the circle of Zinn–Haller [16,18]. In myopic eyes, optic disc tilt, misalignment between the BMO and the clinically visible disc margin, enlargement of the PPA region, and thinning of the peripapillary sclera and choroid may alter the anatomical relationships of these deep vascular structures [21,54]. An LSFG study comparing normal eyes and eyes with myopic optic neuropathy (MON) also suggested that tissue-area mean blur rate (MBR-T) is reduced in MON [38]. However, because studies specifically examining deep optic nerve head circulation in MON alone remain limited, further investigation is needed.

### 5.4. Peripapillary Choroidal Circulation

The choroid is a highly perfused tissue supplied primarily by the SPCAs and plays a critical role in delivering oxygen and nutrients to the outer retina and retinal pigment epithelium [15,16]. In myopic eyes, particularly highly myopic eyes, choroidal thinning has been consistently reported in association with stretching of posterior pole tissues caused by axial elongation [55]. In pathologic myopia, choroidal thinning may be accompanied by diffuse chorioretinal atrophy, patchy atrophy, and posterior staphyloma, which may lead to heterogeneous alterations in the choroidal vascular architecture and blood flow distribution [56,57,58].

Around the optic nerve head, the peripapillary choroid is supplied by the SPCA system, and it is anatomically adjacent to the deep optic nerve head circulation, including the lamina cribrosa and the circle of Zinn–Haller [18,23]. Therefore, thinning of the peripapillary choroid and alterations in its vascular architecture associated with high myopia may also influence the perfusion environment of the deep optic nerve head [21,54]. Although choroidal blood flow has been suggested to be involved in the regulation of ocular growth in pediatric myopia [59,60,61], it remains unclear whether reduced choroidal circulation precedes impairment of optic nerve head circulation in adult eyes with high myopia. These changes should therefore be interpreted as interrelated alterations associated with myopic posterior pole remodeling, rather than as a simple causal sequence [62].

## 6. Glaucoma-Related Vascular Changes

In glaucoma, in addition to damage to retinal ganglion cells and their axons, reduced flow-related parameters in the optic nerve head and peripapillary region have been widely reported. Whether ocular blood flow reduction is a cause of GON, a secondary change resulting from neural tissue damage, or a change that progresses in parallel with neural damage remains incompletely understood. However, with the increasing use of OCTA and LSFG, it has become evident that glaucomatous eyes show reduced flow-related parameters at multiple levels, including the superficial retinal circulation, device-defined deep peripapillary slabs, and the optic nerve head tissue measured by LSFG.

In this section, we summarize representative blood flow alterations associated with GON, including reduced RPC vessel density, CMvD, and reduced optic nerve head tissue blood flow measured by LSFG, and we discuss their relationship with lamina cribrosa deformation. Table 1 summarizes the structural and vascular characteristics of MON and GON, including overlapping and uncertain findings.

### 6.1. Reduction in Peripapillary Retinal Microvasculature

OCTA studies have reported reduced vessel density in the peripapillary retina of glaucomatous eyes, particularly in the RPCs [63]. RPCs mainly reflect the superficial retinal circulation derived from the central retinal artery and run closely along the RNFL. Therefore, reductions in RPC vessel density often show spatial correspondence with glaucomatous RNFL thinning and visual field defects [64]. Liu et al. visualized reduced peripapillary perfusion in glaucomatous eyes using OCTA and demonstrated that vessel density and an index of peripapillary flow were useful for differentiating glaucomatous eyes from normal eyes [65].

However, reduced RPC vessel density does not by itself necessarily indicate a primary vascular impairment. Because RPCs run within the RNFL, vessel density may decrease secondarily in association with RNFL thinning [66]. OCTA-derived parameters are also affected by signal strength and segmentation errors [67]. Thus, reduced superficial peripapillary vessel density may reflect both secondary changes associated with neural tissue loss and microcirculatory impairment. Future longitudinal studies integrating structural, vascular, and functional assessments are needed to clarify the temporal significance of RPC loss in glaucoma [68].

### 6.2. Choroidal Microvasculature Dropout

In glaucomatous eyes, CMvD is generally described as a focal sectoral absence of or marked reduction in flow-related signal within a device-defined deep peripapillary slab, typically adjacent to the optic disc margin (juxtapapillary location) and often within the β-zone PPA [26,69]. Kim et al. described flow-signal voids in a non-juxtapapillary location, typically in the distal PPA or γ-zone of healthy myopic eyes; such lesions should not be classified as glaucomatous CMvD [48]. CMvD may reflect localized perfusion impairment in the peripapillary choroid or deep optic nerve head circulation. Studies comparing OCTA with ICGA have reported that CMvD detected by OCTA corresponds to choroidal filling defects on ICGA, suggesting that CMvD is not merely an OCTA artifact but may represent an actual deep perfusion defect [70,71].

In glaucomatous eyes, CMvD has been reported to be associated with retinal nerve fiber layer defects [72], optic disc hemorrhage [73,74], lamina cribrosa defects [75], central visual field damage [76], and disease progression [77]. Longitudinal studies have further shown that CMvD can enlarge over time and that eyes with CMvD have a higher risk of glaucoma progression [77]. Therefore, CMvD may serve as an imaging biomarker associated with local structural and functional damage in GON.

However, the mechanisms underlying CMvD formation and the causal relationship between CMvD formation and glaucomatous damage remain incompletely understood. It is still unclear whether CMvD precedes neural damage, develops secondarily to lamina cribrosa deformation or retinal nerve fiber layer thinning, or progresses in parallel with local structural vulnerability and vascular impairment. In addition, Akagi et al. reported that some findings that appear similar to MvD in OCTA en face images might be pseudo-MvD, highlighting the need to confirm its presence in B-scan images and segmentation lines [78].

### 6.3. Relationship Between CMvD and Lamina Cribrosa Deformation

The lamina cribrosa is considered a principal site of damage in GON, and OCT can visualize focal lamina cribrosa defects and deformation [79,80]. OCTA studies have reported that CMvD is frequently observed in glaucomatous eyes with focal lamina cribrosa defects [75]. Reduced OCTA vessel density and spatial correspondence between lamina cribrosa defects and CMvD have also been described [75,81].

These findings support a spatial association between focal lamina cribrosa abnormalities and deep peripapillary flow-signal deficits. Because most of the available evidence is cross-sectional, it remains uncertain whether structural deformation precedes the vascular finding, the vascular finding precedes structural damage, or both reflect a shared local susceptibility. Lamina cribrosa defects and CMvD may therefore be interpreted as paired structural and perfusion-related biomarkers, without assigning a causal direction.

### 6.4. Optic Nerve Head Blood Flow Measured by LSFG

MBR-T in the optic nerve head tissue area has been reported to be reduced in glaucomatous eyes, and this reduction is associated with RNFL thinning and the severity of visual field damage [38]. Our group has published a series of reports on glaucomatous optic nerve head blood flow measurements made using LSFG. Shiga et al. compared normal eyes, eyes with preperimetric glaucoma, and eyes with early NTG and showed that MBR-T was already reduced at the stage of preperimetric glaucoma [37]. We also used LSFG waveform analysis to demonstrate that, in NTG, pulsatility and waveform characteristics are altered, suggesting that waveform parameters, in addition to MBR, may be useful for evaluating ocular blood flow in glaucoma [35].

Furthermore, in a prospective study of eyes with preperimetric glaucoma, Shiga et al. reported that baseline optic nerve head tissue blood flow may predict subsequent visual field progression [82]. Kiyota et al. also demonstrated sectoral differences in the relationship between optic nerve head blood flow and corresponding visual field damage, suggesting that localized reduction in optic nerve head blood flow may be associated with the topography and progression of visual field defects [83]. These studies suggest that optic nerve head blood flow is an important biomarker in glaucomatous eyes.

**Table 1:** Structural and vascular characteristics of MON and GON, including overlapping or uncertain findings.

Parameter	MON-Dominant Findings	GON-Dominant Findings	Overlapping or Uncertain Findings
Optic disc tilt/torsion	Common in axial elongation and posterior-pole remodeling [42,44,45].	Not GON-specific; may modify sectoral damage in myopic glaucoma [84,85].	May mimic GON and increase OCT/OCTA measurement error [30,42,86].
β-/γ-zone PPA	γ-zone lacks Bruch’s membrane and reflects myopic expansion/BMO misalignment [46,47,54].	β-zone may accompany GON and is the typical CMvD location [26,46,70].	Zones may coexist; assess structural boundaries and lesion location [42,46,48,54].
RNFL changes	Axial length, tilt, and segmentation can cause atypical or apparent thinning [30,42,43].	Reproducible sectoral thinning/progression with visual-field loss [80,87,88,89].	Confirm equivocal findings with longitudinal OCT and visual fields [30,86].
RPC vessel density (OCTA)	Magnification-sensitive; reductions may occur in pathologic myopia [27,51,52,53].	Lower density is spatially associated with RNFL and visual-field loss [63,64,65,66,67,68].	Not volumetric flow; artifacts and secondary rarefaction remain possible [25,28,29].
Peripapillary MvD/CMvD (OCTA)	Non-juxtapapillary MvD may occur in distal PPA or γ-zone without glaucoma [48].	Device-defined deep-slab CMvD is associated with local GON damage/progression [70,72,73,75,76,77].	Assess location, β-/γ-zone anatomy, segmentation, and B-scans; causality is unproven [28,48,69,75,78].
Lamina cribrosa abnormalities	Deformation may accompany axial elongation and disc remodeling [44,54,90].	Thinning or focal defects are associated with RNFL loss and CMvD [75,79,80,81].	Spatial association does not establish temporal order [75,81].
Visual-field characteristics	Nonspecific defects; some remain stable [42,85,86].	Arcuate, nasal, or paracentral defects with reproducible progression [88,89,91,92].	Patterns overlap; longitudinal and central 10-2 testing aid interpretation [88,89,91,92].

BMO, Bruch’s membrane opening; CMvD, choroidal microvasculature dropout; GON, glaucomatous optic neuropathy; MON, myopic optic neuropathy; MvD, microvasculature dropout; OCT, optical coherence tomography; OCTA, optical coherence tomography angiography; PPA, peripapillary atrophy; RNFL, retinal nerve fiber layer; RPC, radial peripapillary capillary.

### 6.5. Flammer Syndrome

In NTG, vascular dysregulation has been implicated as an IOP-independent mechanism [10]. Flammer syndrome, a clinical phenotype of primary vascular dysregulation, is characterized by systemic features such as low blood pressure, cold extremities, increased sensitivity to cold, and a vasospastic tendency [93]. Flammer syndrome-related findings have been reported more frequently in patients with NTG, supporting a potential link between systemic vascular dysregulation and impaired ocular blood flow regulation [94,95]. In these eyes, unstable ocular perfusion, impaired autoregulation, and repeated fluctuations in optic nerve head blood flow may increase the susceptibility to glaucomatous damage [96].

## 7. Ocular Blood Flow Vulnerability in Eyes with Myopia and Glaucoma

In eyes with myopia and glaucoma, myopia-related structural deformation and glaucoma-associated perfusion abnormalities may overlap. Axial elongation and peripapillary remodeling can alter the anatomical relationship between the optic nerve head and its vascular supply, potentially modifying the location and severity of glaucomatous blood flow impairment. Myopic structural changes should therefore be regarded as potential modifiers of the location and interpretation of perfusion-related findings, rather than as established amplifiers of vascular injury. Table 2 summarizes the principal OCTA- and LSFG-derived biomarkers, their confounders, and their current level of evidence.

### 7.1. Myopia-Related Anatomical Changes Relevant to Deep Optic Nerve Head Perfusion

In eyes with myopia and glaucoma, axial elongation and posterior-pole stretching alter the spatial relationships among the optic nerve head, lamina cribrosa, peripapillary sclera, choroid, and SPCA-derived circulation [84,90,97,98]. These anatomical changes provide a plausible context for altered flow-related measurements and sectoral correspondence with neural damage. However, current longitudinal evidence does not demonstrate that myopic deformation produces a reduced vascular reserve or increases susceptibility to glaucomatous injury. Myopic anatomy should therefore be treated as a potential modifier and source of measurement complexity, rather than an established causal vascular mechanism.

### 7.2. Cause, Consequence, or Parallel Change?

It remains incompletely understood whether reduced ocular blood flow in glaucomatous eyes is a cause of neural damage, a secondary consequence of neural tissue loss, or a change that progresses in parallel with neurodegeneration. For example, Kamalipour et al. reported that, in early primary open-angle glaucoma, the degree of RNFL thinning and reduction in vessel density varied among individual cases [87]. In contrast, CMvD and reduced optic nerve head tissue blood flow measured by LSFG may reflect deeper peripapillary circulatory impairment and, in some cases, may represent a pathological process that precedes or progresses in parallel with neural damage [77,82,99].

Kiyota et al. investigated the temporal relationship between reduced optic nerve head blood flow and RNFL thinning in eyes with open-angle glaucoma and showed that, depending on patient characteristics and the location of damage, blood flow reduction may precede structural damage, whereas structural damage may precede blood flow reduction in other cases [100]. In particular, they reported that reduced optic nerve head blood flow may be more likely to precede RNFL thinning in eyes with risk factors such as older age, higher heart rate, and damage in the superotemporal sector [100].

These findings suggest that vascular impairment in glaucoma does not have a uniform meaning, but may appear as a cause, consequence, or parallel change depending on the individual case and affected sector. From this perspective, eyes with GON and myopia may therefore represent a group in which perfusion-related findings are more readily detected, although this does not establish that vascular impairment drives progression. Conversely, in myopic eyes, secondary reductions in vascular parameters may also occur as a result of neural tissue loss or optic disc morphological changes. Therefore, in the combination of myopia and glaucoma, reduced blood flow should not be interpreted simply as either a cause or a consequence. Rather, it should be understood as part of a dynamic process in which structural deformation, neural damage, and vascular impairment are mutually interrelated.

### 7.3. Sectoral Vulnerability and Central Visual Field Damage

In patients with GON and myopia, vascular impairment should be evaluated not only as a global reduction but also as a sectoral phenomenon. Optic disc tilt and torsion in myopic eyes may impose asymmetric mechanical stress on peripapillary tissue [42,85,101]. Sectoral perfusion-related abnormalities, structural damage, and visual-field defects may be observed in corresponding regions of myopic glaucomatous eyes [83,84].

The macular vulnerability zone proposed by Hood et al. provides an important framework for understanding central visual field damage [88,89]. The inferotemporal optic nerve head contains fibers projecting toward the macular and paracentral regions; therefore, damage in this sector can affect central visual function. In myopic eyes, disc tilt, torsion, CMvD, reduced peripapillary flow-related signal, and paracentral loss have been reported in overlapping sectors [14,91,102]. These cross-sectional correspondences support targeted central-field assessment but do not show that the perfusion abnormality amplifies or causes visual-field loss.

Indeed, central visual field damage and visual acuity loss have been reported as clinically important issues in glaucomatous eyes with myopia. In the classification of glaucomatous optic disc morphology proposed by Nicolela et al. [103], the myopic glaucomatous disc type has been shown to be associated with characteristic patterns of visual dysfunction [92,104], and Chihara et al. also reported that central visual field damage may be more frequent in myopic eyes [105]. Consistent with this, Kiyota et al. assessed the PPA zone with OCTA in eyes with NTG and a myopic disc, and reported that the choroidal signal intensity within this zone was associated with axial elongation and central retinal structural and functional measures [106]. In addition, baseline temporal optic nerve head and PPA-zone blood flow measured by LSFG was associated with the severity and progression of central visual field damage in eyes with open-angle glaucoma and a myopic disc [107]. Therefore, in patients with GON and myopia, assessment with 10-2 perimetry and central visual field indices should be considered in addition to conventional 24-2 perimetry.

**Table 2:** Principal OCTA- and LSFG-derived vascular biomarkers: associations, confounders, evidence, and selected references.

Modality/Parameter	Reported Association	Main Confounders	Clinical Significance/Evidence
OCTA—RPC vessel density	Lower in GON; myopic estimates are magnification-sensitive [27,52,63,65].	Magnification, disc tilt, segmentation, signal strength, and device effects [27,28,29,108].	Cross-sectional correlate with limited longitudinal evidence; not direct flow or a causal marker [25].
OCTA—CMvD/non-juxtapapillary MvD	CMvD correlates with focal GON damage/progression; non-juxtapapillary MvD can occur without glaucoma [48,72,77].	Slab definition, lesion location, artifacts, segmentation, and B-scan confirmation [26,28,48,78].	Potential focal/prognostic biomarker; causality and treatment utility remain unproven [73,77,99].
OCTA—pulsatile flow signal	Time-dependent flow-signal changes occur with markedly elevated IOP [31,32].	Cardiac cycle, acquisition timing, IOP, motion, threshold, and device algorithms [25,28].	Preliminary physiological finding; not a validated biomarker [31,32].
LSFG—MBR-T and waveform indices	Lower MBR-T and altered waveforms are reported in GON, including sectoral/prognostic associations [35,37,38,82,83,107].	IOP, blood pressure, heart rate, refraction/axial length, disc morphology, and imaging conditions [39].	Repeatable relative hemodynamic measure; has not been validated as a treatment surrogate [33,34,82,100].

CMvD, choroidal microvasculature dropout; GON, glaucomatous optic neuropathy; IOP, intraocular pressure; LSFG, laser speckle flowgraphy; MBR-T, tissue-area mean blur rate; MvD, microvasculature dropout; OCTA, optical coherence tomography angiography; RPC, radial peripapillary capillary.

## 8. Potential Blood Flow-Improving Treatments

IOP reduction remains the most established therapeutic intervention for glaucoma, including in eyes with coexisting myopia. IOP reduction may not only alleviate mechanical stress but also secondarily improve optic nerve head blood flow by increasing ocular perfusion pressure and reducing compression around the lamina cribrosa. For example, studies comparing ocular fundus blood flow before and after trabeculectomy have reported increases in ocular blood flow and in flow stability after IOP reduction [109,110].

Topical agents such as tafluprost, brimonidine, and Rho-associated protein kinase inhibitors have shown ocular blood-flow or related functional effects in selected studies [111,112,113,114,115,116,117]. Among these, only brimonidine has been evaluated with randomized visual-field outcomes [113,114]. The evidence is heterogeneous and is not specific to highly myopic glaucoma. β-blockers may also influence nocturnal systemic blood pressure and perfusion [118,119,120]. Drug selection should therefore remain based primarily on proven IOP-lowering efficacy, tolerability, and systemic safety rather than on a presumed blood-flow benefit.

Red-ginger-based supplements and black currant anthocyanins have preliminary experimental or limited clinical evidence for changes in ocular blood flow [121,122,123]. No evidence currently supports their routine use specifically for myopic glaucoma, and confirmation in adequately powered trials with structural and visual-field outcomes is required.

## 9. Limitations of Current Research

This review has summarized findings from studies using OCTA-derived perfusion-related parameters and LSFG-derived hemodynamic indices in myopia and glaucoma. Although numerous studies have established myopia as a risk factor for glaucoma and have suggested possible vascular mechanisms, many of these studies are cross-sectional. Therefore, the temporal relationship among myopia, altered ocular circulatory parameters, and glaucoma progression remains incompletely understood [68].

In addition, many studies have relied on OCTA, but its interpretation requires caution. OCTA imaging algorithms differ among devices, and even in the same eye, vascular images may vary depending on the OCTA system used to obtain them [124,125,126]. Therefore, results from different OCTA studies may not be directly comparable [108]. Moreover, OCTA-based blood flow assessment depends heavily on image quality and is susceptible to various artifacts [28,29]. In MON, structural alterations around the optic nerve head may further increase the likelihood of artifacts [30]. Consequently, segmentation errors and signal attenuation are more likely to occur, and further studies are needed to determine whether reduced vessel density truly reflects circulatory insufficiency in myopic eyes.

Ocular blood flow is strongly influenced not only by myopia and glaucoma but also by a variety of systemic and ocular factors, including systemic blood pressure, ocular perfusion pressure, diabetes mellitus, dyslipidemia, use of systemic medications, and autonomic nervous system function. Previous reviews have emphasized the importance of systemic blood pressure and ocular perfusion pressure in glaucoma, and autonomic dysfunction has also been linked to altered ocular blood flow [127]. However, many studies have not been able to fully account for all of these potential confounders. Therefore, it remains difficult to isolate the specific contributions of myopia or glaucoma themselves to reduced ocular blood flow.

In addition, accurate differentiation between MON and GON remains challenging. High myopia can remodel the optic nerve head and peripapillary tissues, producing structural and functional abnormalities that may mimic glaucomatous damage, and recent reviews have highlighted the diagnostic difficulty of evaluating glaucoma in myopic eyes [86]. As a result, study populations may differ substantially across reports, and the possibility that MON and GON overlap within individual cohorts cannot be excluded.

## 10. Conclusions

Although both MON and GON may be accompanied by reduced ocular blood flow, the clinical significance and underlying mechanisms differ between these conditions. Myopia and glaucoma frequently coexist, and the anatomical background of MON may complicate the interpretation of perfusion-related findings in GON. Current evidence, however, does not establish whether these perfusion abnormalities are causal, consequential, or parallel to neurodegeneration. As the global prevalence of myopia continues to increase, the burden of combined myopia and glaucoma is also expected to rise. Although evidence-based treatments beyond IOP reduction have not yet been established, a better understanding of the interaction between myopia-induced mechanical stress and glaucoma-associated vascular impairment may provide a foundation for developing more individualized therapeutic strategies tailored to the underlying pathophysiology.

## Data Availability

No new data were created or analyzed in this study. Data sharing is not applicable to this article.

## Abbreviations

The following abbreviations are used in this manuscript:

BMO	Bruch’s membrane opening
BM–RPE	Bruch’s membrane–retinal pigment epithelium
CMvD	choroidal microvasculature dropout
CRA	central retinal artery
GON	glaucomatous optic neuropathy
ICGA	indocyanine green angiography
IOP	intraocular pressure
LSFG	laser speckle flowgraphy
MBR	mean blur rate
MBR-T	tissue-area mean blur rate
MON	myopic optic neuropathy
MvD	microvasculature dropout
NTG	normal-tension glaucoma
OCT	optical coherence tomography
OCTA	optical coherence tomography angiography
PCA	posterior ciliary artery
PPA	peripapillary atrophy
RNFL	retinal nerve fiber layer
RPC	radial peripapillary capillary
SPCA	short posterior ciliary artery
